# World TB Day —March 24, 2018

**DOI:** 10.15585/mmwr.mm6711a1

**Published:** 2018-03-23

World TB Day is recognized each year on March 24, commemorating the date in 1882 when Dr. Robert Koch announced his discovery of *Mycobacterium tuberculosis*, the bacillus that causes tuberculosis (TB). World TB Day is an opportunity to raise awareness about TB and the measures needed to tackle this devastating disease. The U.S. theme for World TB Day 2018, “Wanted: leaders for a TB-free United States. We can make history. End TB” highlights the importance of engaging and empowering public health partners, clinicians, and communities in efforts to eliminate TB.

A study reported in this issue of *MMWR* found that in 2017, a provisional total of 9,093 TB cases were reported in the United States (rate of 2.8 cases per 100,000 persons) (*1*), a decrease from the 2016 case count and rate and the lowest rate and number of TB cases on record since reporting began in 1953. However, increased diagnosis and treatment of latent TB infection is important for eliminating TB in the United States (*2*).

CDC is working to eliminate TB in the United States by engaging new domestic and global partners, strengthening control of TB disease, and expanding testing and treatment of latent TB infection. Additional information about World TB Day and CDC’s TB elimination activities is available online (https://www.cdc.gov/tb/worldtbday).
